# Differences in the sensitivity of classically and alternatively activated macrophages to TAK1 inhibitor-induced necroptosis

**DOI:** 10.1007/s00262-020-02623-7

**Published:** 2020-05-29

**Authors:** Zsófia Varga, Tamás Molnár, Anett Mázló, Ramóna Kovács, Viktória Jenei, Krisztina Kerekes, Attila Bácsi, Gábor Koncz

**Affiliations:** 1 Department of Immunology, Faculty of Medicine, University of Debrecen, 1 Egyetem Square, Debrecen, 4032 Hungary; 2 Doctoral School of Molecular Cellular and Immune Biology, University of Debrecen, Debrecen, Hungary; 3 MTA-DE Cell Biology and Signalling Research Group, University of Debrecen, Debrecen, Hungary; 4 BBS Nanotechnology Ltd., Debrecen, Hungary

**Keywords:** Necroptosis, Macrophage, Inflammation, Cancer

## Abstract

Controlling the balance of pro-inflammatory M1 versus anti-inflammatory M2 macrophages may have paramount therapeutic benefit in cardiovascular diseases, infections, cancer and chronic inflammation. The targeted depletion of different macrophage populations provides a therapeutic option to regulate macrophage-mediated functions. Macrophages are highly sensitive to necroptosis, a newly described regulated cell death mediated by receptor-interacting serine/threonine-protein kinase 1 (RIPK1), RIPK3 and mixed lineage kinase domain like pseudokinase. Antagonists of inhibitors of apoptosis proteins (SMAC mimetics) block RIPK1 ubiquitination, while TGF-activated kinase 1 (TAK1) inhibitors prevent the phosphorylation of RIPK1, resulting in increased necroptosis. We compared the sensitivity of monocyte-derived human M1 and M2 cells to various apoptotic and necroptotic signals. The two cell types were equally sensitive to all investigated stimuli, but TAK1 inhibitor induced more intense necroptosis in M2 cells. Consequently, the treatment of co-cultured M1 and M2 cells with TAK1 inhibitor shifted the balance of the two populations toward M1 dominance. Blockage of either Aurora Kinase A or glycogen synthase kinase 3β, two newly described necroptosis inhibitors, increased the sensitivity of M1 cells to TAK1-inhibitor-induced cell death. Finally, we demonstrated that in vitro differentiated tumor-associated macrophages (TAM-like cells) were as highly sensitive to TAK1 inhibitor-induced necroptosis as M2 cells. Our results indicate that at least two different necroptotic pathways operate in macrophages and the targeted elimination of different macrophage populations by TAK1 inhibitor or SMAC mimetic may provide a therapeutic option to regulate the balance of inflammatory/anti-inflammatory macrophage functions.

**Electronic supplementary material:**

The online version of this article (10.1007/s00262-020-02623-7) contains supplementary material, which is available to authorized users.

## Introduction

Macrophages with highly polarized functions coexist in tissues throughout the body to ensure the modulation of immune responses. Traditionally, macrophages can be subdivided into classically activated M1 and alternatively activated M2 phenotypes. M1 cells provide the first line of immune defense and activate both innate and adaptive immunity, while M2 macrophages are responsible for the regulation of tissue regeneration, are involved in the clearance of apoptotic bodies and contribute to the immune suppression [1]. Controlling the balance of pro-inflammatory versus anti-inflammatory macrophages may have paramount therapeutic benefit in all the world’s leading causes of death, such as in cardiovascular diseases [2, 3], infections [4], cancers [5, 6], chronic inflammation [7], diabetes [8] or autoimmune reactions [9].

The success of immunotherapy highlights the effectiveness of the immune system in tumor eradication. Tumors still develop in spite of the immune attack, because tumors are surrounded by immunosuppressive cells and can escape from immune surveillance by hampering the onset of an effective anti-tumor immune response [10]. The tumor microenvironment (TME) consists of various immune cells, where macrophages form one of the most abundant cell populations. Solid tumors manipulate macrophage recruitment and regulate macrophage differentiation. Reprogrammed tumor-associated macrophages (TAM) supports tumor formation through upregulation of angiogenesis, growth factor production or immunosuppression, and these cells also promote metastasis and increase drug resistance [5, 6], Unlike monocytes, macrophages have a long life span of months to years [11]. Accordingly, macrophages are relatively resistant to most apoptotic stimuli, but are highly sensitive to two newly described inflammatory forms of regulated cell death, necroptosis [12, 13] and pyroptosis [14]. Necroptosis is a regulated event rather than an accidental cell death process in which the most critical contributors are receptor-interacting protein 1 (RIPK1) [15], RIPK3 [16] and mixed lineage kinase domain like pseudokinase (MLKL) [17]. Necroptosis is known to play an important role in the pathogenesis of many diseases, such as neurodegenerative or inflammatory disorders, gastrointestinal, cardiovascular and pulmonary diseases [18]. The critical receptors of macrophages such as death, pattern recognition, DNA binding, cytokine and adhesion receptors all have been identified as potential inducers of necroptosis [19]. Necroptosis can be activated when apoptosis is blocked and pro-necroptotic proteins are released from caspase-8-mediated inhibition [15]. Active caspase-8 blocks the necroptotic mode [15] of action preferentially through the cleavage of RIPK1 [20], RIPK3 [21] and the cylindromatosis (CYLD) protein, which mediates deubiqutination of RIPK1 [22]. The ubiquitination of RIPK1 by inhibitors of apoptosis proteins (cIAPs) initiates cell survival [23]. The created ubiquitin network allows the activation of TGF-activated kinase 1 (TAK1), which mediates survival signals by (1) activating the NFκB and MAPK signaling pathways and thereby increasing the expression of several prosurvival molecules [24, 25], (2) preventing the interaction between RIPK1 and cell death-related molecules [26], (3) regulating RIPK1 phosphorylation directly [27] or indirectly by activating I kappa B *kinases* (IIKKα/IKKβ) [28] or mitogen-activated protein kinase-activated protein kinase 2 (p38MAPK/MK2) [29]. In addition to TAK1- and cIAP-mediated downregulation, more than 70 molecules play a role in the regulation of necroptosis [18], among them Aurora kinase A (AURKA), which interacts directly with RIPK1 and RIPK3 in nontreated cells to reduce unwanted necroptosis [30]. Its downstream target glycogen synthase kinase 3β (GSK3β) regulates the formation of the necrosome and suppresses necroptosis [30]. In the absence of ubiquitylation and/or phosphorylation, RIPK1 transduces cell death signals, and when apoptotic pathways are blocked, necroptosis is activated. Thus, necroptosis is most frequently induced in in vitro experimental systems by utilizing pan caspase inhibitors in combination either with IAP antagonists, termed SMAC mimetics (SM) to block RIPK1 ubiquitination [12], or with TAK1 inhibitors to prevent the phosphorylation of RIPK1 [13]. Necroptotic cell death of macrophages has already been shown following treatment with SM [31] or TAK1 inhibitors [14].

Many clinical trials aim to modify the M1/M2 ratio, but currently, the targeted depletion of a unique macrophage subtype by specific cell death signals is not a therapeutic option. We aimed to identify circumstances in which M2 cells or TAMs are susceptible to cell death signals, but M1 cells remain resistant. We found that M2 macrophages were highly sensitive, while M1 macrophages were unaffected by TAK1 inhibitor-generated necroptosis. The resistant M1 macrophages harness AURKA-mediated inhibition in the downregulation of cell death. In contrary to TAK1 inhibitor, SM treatment results in necroptosis in both macrophage populations, highlighting that at least two different necroptotic pathways operate in macrophages. TAK1 inhibitor-induced necroptosis pushes the ratio of M1/M2 macrophages toward an inflammatory phenotype, which rationalizes the activation of necroptosis for therapeutic intervention in any disease where M1 functions are preferred.

## Materials and methods

### Antibodies and reagents

The following commercial antibodies and reagents were used in this study: Z-VAD, AURKA inhibitor CCT137690, MAPK inhibitors SB203508-p38, U0126-ERK, NFκB inhibitor-TPCA1-IKK were purchased from ApexBio, GSK3ß inhibitor AR-A014418 was from Selleck Chemicals, and SP600125-JNK was from Santa Cruz. TNF alpha was purchased from PeproTech. 5Z-7-oxozeaenol (5Z-7) and RIPK3 inhibitor (GSKʹ872) were from Sigma-Aldrich. Necrostatin-1 was from Abcam, and TNF-R1:Fc fusion protein was from Adipogen. Lipopolysaccharide (LPS) was from InvivoGen, and Birinapant was from LC Laboratories. The flow cytometry antibodies were purchased from the following companies: CD209-PE (DC-SIGN, BioLegend), CD206-Pe-Cy^TM^5 (BD Pharmingen) and CD80-FITC (SONY Biotechnology), CD14-PE (BioLegend), HLA-DR-PercP (BD Pharmingen), PD-1/CD279-PercP (BioLegend), CD163-PE (Biosciences).

### Generation of monocyte-derived M1, M2 macrophages and TAM-like cells

Heparinized leukocyte-enriched buffy coats were obtained from healthy blood donors, and peripheral blood mononuclear cells (PBMCs) were separated from buffy coats by Ficoll-Paque Plus (Biosciences) gradient centrifugation. Monocytes were purified from PBMCs by positive selection using immunomagnetic cell separation and anti-CD14-conjugated microbeads (Miltenyi Biotec), according to the manufacturer’s instructions. After separation on a VarioMACS magnet, 96–99% of the cells were shown to be CD14^+^ monocytes.

Isolated monocytes were cultured for 5 days in 6-well tissue culture plates at a density of 2.0 × 10^6^ cells/ml in Gibco’s serum-free AIM-V medium (Thermo Fischer Scientific) supplemented with 50 ng/ml M-CSF (PeproTech). In order to acquire the M1 and M2 types, cells were stimulated on the fifth day of differentiation for 24 h with lipopolysaccharide (50 ng/ml ultrapure LPS, InvivoGen), IFNγ (20 ng/ml, PeproTech) to M1 and IL-4 (20 ng/ml, PeproTech), IL-10 (20 ng/ml, PeproTech) and TGFß (20 ng/ml, PeproTech) to M2 phenotype. For the differentiation of TAM-like cells, isolated monocytes were cultured for 5 days in 6-well tissue culture plates at a density of 2.0 × 10^6^ cells/ml in Thp-1 supernatant supplemented with IL-4 (20 ng/ml), IL-10 (20 ng/ml) and TGFß (20 ng/ml). On the fifth day, TAM-like cells were treated again with Thp-1 supernatant for 24 h.

### Production of THP-1 supernatant

To generate THP-1 supernatant, cells were cultured at a density of 2 × 10^5^ cells/ml in Gibco’s serum-free AIM-V medium (Thermo Fischer Scientific) for 2 days and the supernatant was collected at 1500 rpm for 5 min.

### Measurement of cytokine concentration

The supernatants of M1 and M2 macrophages were harvested on the sixth day of differentiation, and the concentrations of IL-12 and IL-10 cytokines were measured using OptEIA kits (BD Biosciences) following the manufacturer’s instructions.

### Flow cytometry

Cell death was induced by TAK1 inhibitor (5Z-7-oxozeaenol 1 µM), IAP antagonist (Birinapant 0.5 µM) LPS (100 ng/ml), TNF alpha (60 ng/ml), caspase inhibitor (Z-VAD 50 µM), RIP1K inhibitor, Necrostatin-1 38.5 μM), RIPK3 inhibitor (GSKʹ872 7.5 μM), AURKA inhibitor (CCT137690 1.25 μM), GSK3β inhibitor (AR-A014418 10 μM) and MAPK (SP600125-JNK 1 µM; SB203580-p38 1 µM; U0126-Erk 1 µM) and NFκB inhibitors (TPCA1-IKK 1 µM) on M1, M2, TAM and M1-M2 co-cultured macrophages.

Total cell death was quantified based on the loss of membrane integrity and the uptake of propidium iodide (PI, Sigma-Aldrich). Cells were stained with PI (10 µg/ml) before analysis by flow cytometry. Cell death was measured by flow cytometry using FACS Calibur (BD Biosciences), and data were analyzed by FlowJo software (Tree Star, Ashland, OR, USA).

### Western blotting

Protein extraction was performed by lysing the cells in 2 × Laemmli sample buffer. Proteins were separated by SDS gel electrophoresis using 10% polyacrylamide gels and transferred onto nitrocellulose membranes ER (Bio-Rad Laboratories). Nonspecific binding was blocked by TBS-Tween with 5% nonfat dry milk. Transfer membranes were immunoblotted with the indicated antibodies: RIPK1 (BD Biosciences), RIPK3 (Cell Signaling), MLKL (Sigma-Aldrich), pMLKL (Cell Signaling), TAK1 (Cell Signaling), GSK3ß (Cell Signaling), Aurora A (Cell Signaling), p38 (Thermo Fischer Scientific), pErk (Thermo Fischer Scientific), pJNK (Thermo Fischer Scientific), pIκß (R&D Systems) and β-actin (Sigma-Aldrich) all diluted 1:1000. Anti-rabbit (GE Healthcare), anti-mouse (GE Healthcare) or anti-rat (Sigma-Aldrich) antibodies conjugated to horseradish peroxidase were used as the secondary antibodies.

### Co-cultures of M1–M2 cells

To test the functional importance of differences in cell death intensity between M1 and M2 cells, a macrophage co-culture was created.

An equal number of M1 and M2 macrophages, 4 × 10^5^ cells/0.5 ml, were co-cultured for 4 h in 24-well tissue culture plates. Mixture of M1 and M2 cells was treated with necroptotic stimuli. The percentage of surviving macrophages was labeled with anti-CD209-PE and anti-CD80-FITC antibodies in 24 h.

To distinguish between M1 and M2 cell death, M2 macrophages were stained using CellTracker™ Green CMFDA Dye (Thermo Fischer Scientific). M2 cells were loaded with 10 ng/ml CellTracker™ Green CMFDA Dye at 37 °C for 30 min. After intense washing, 4x10^5^ cells in 0.5 ml volume were co-cultured with unlabeled M1, in the same concentration in 24-well tissue culture plates. M1 and M2 co-cultures were treated with necroptotic stimuli, and the cell death of the different populations was determined after 24 h by PI staining.

### Statistical analysis

Two-way ANOVA or one-way ANOVA followed by *Sidak’s multiple-comparison test* was used for multiple comparisons. Results are expressed as mean ± SD. In the case of IL10 and IL-12 production, significance was calculated by Student’s *t* test. The results are expressed as mean +SD. All analyses were performed by using GraphPad Prism software, version 6.0. Differences were considered to be statistically significant at *P* < 0.05. Significance is indicated by *P < 0.05; **P < 0.01; ***P < 0.005.

## Results

### M2 macrophages, but not M1 cells are sensitive to TAK1 inhibitor-induced necroptosis

**Fig. 1:**
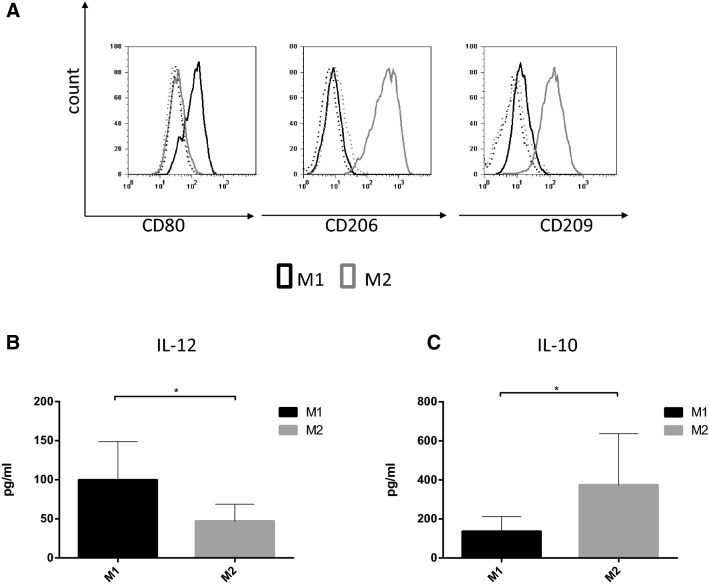
In vitro differentiation of M1 and M2 macrophages. **a** Human monocyte-derived macrophages were differentiated to M1 and M2 phenotype in the presence of LPS (50 ng/ml) and IFNγ (20 ng/ml) or IL-4 (20 ng/ml), IL-10 (20 ng/ml) and TGFß (20 ng/ml), respectively. The cell surface expressions of CD80, CD206 and CD209 were measured on the two cell types by flow cytometry. Representative images of five independent experiments are shown. **b**, **c** The IL-12 and IL-10 production was measured by ELISA. The figure shows the average of three independent experiments

Controlling the balance of pro-inflammatory versus anti-inflammatory macrophages may provide immediate therapeutic benefit. As a new concept for therapeutic intervention, we compared the sensitivity of human M1 and M2 macrophages to various cell death stimuli. For this, blood-derived CD14+ monocytes were separated and differentiated, in accordance with previous studies, in the presence of M-CSF for five days to M0 macrophages and then polarized toward the M1 and M2 cells by stimulation for 24 h with LPS/IFNγ or IL-4/TGFβ, respectively [32]. To confirm the polarization of subtypes, we analyzed cell surface marker expression on the differentiated macrophages. We detected the induction of CD80 on M1 cells, and higher expression level of prototypical M2 markers CD206 and CD209 on M2 cells (Fig. 1a). We also checked the functionality of the two cell populations by measuring the production of M1- and M2-related cytokines. According to widely used protocols, M1 cells released significantly higher amounts of IL-12 than the M2 population, but IL-10 production was more relevant in M2 macrophages (Fig. 1b, c) [33].

**Fig. 2:**
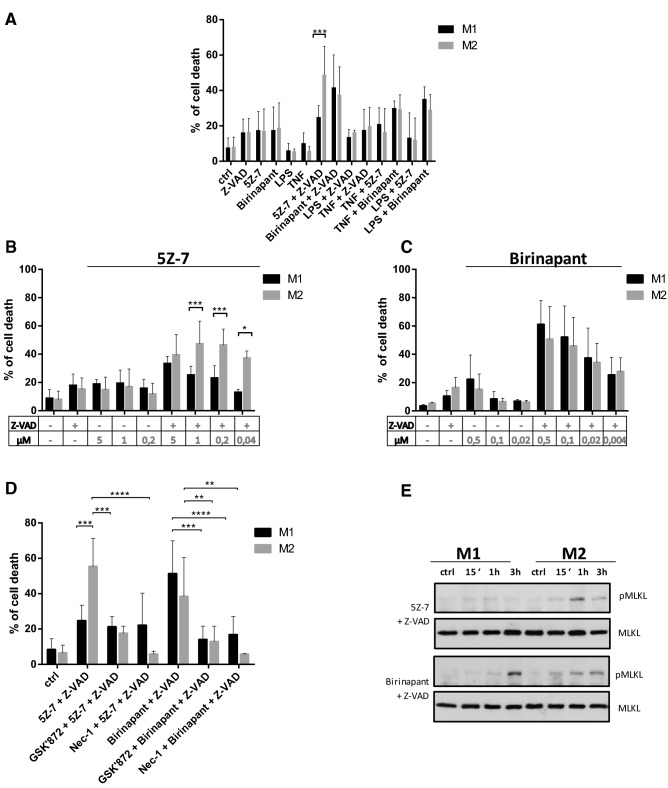
TAK1 inhibitor induces necroptosis on M2, but not on M1 cells. **a** The in vitro differentiated human M1 and M2 cells were stimulated with 100 ng/ml LPS, 60 ng/ml TNF, 0.5 μM birinapant and 1 μM 5Z-7-oxozeaenol with and without 50 μM Z-VAD. **b**, **c** Cells were stimulated with the indicated dose of or 5Z-7-oxozeaenol or birinapant in the presence or absence of 50 μM Z-VAD. **d** Cells were pretreated with 7.5 μM GSKʹ872 RIPK3 inhibitor and 38.5 μM Necrostatin-1 for 1 h, after which 0.5 μM birinapant or 1 μM 5Z-7-oxozeaenol together with 50 μM Z-VAD was added. After 24 h, cell death was determined by PI staining. Figures show the mean plus SD of at least three independent experiments. **e** MLKL phosphorylation (S358) was detected by WB following 0.5 μM birinapant or 1 μM 5Z-7-oxozeaenol treatment for the indicated times. A representative image of three independent experiments

To check the susceptibility to cell death stimuli of polarized M1 and M2 macrophages, we treated these cell types with various apoptotic and necroptotic activators. We used LPS, TNF, SMAC mimetic (birinapant), TAK1 inhibitor (5Z-7-oxozeaenol; 5Z-7) and the combinations of these treatments as apoptotic triggers. In the presence of a caspase inhibitor (Z-VAD), all these stimulants are well-known activators of necroptosis in macrophages [14, 31]. All the investigated stimuli induced cell death at the same level in both cell populations, but TAK1 inhibitor-induced necroptosis was significantly higher in M2 cells (Fig. 2a).

To confirm this result, we studied the dose dependence of birinapant- and 5Z-7-mediated cell death. 5Z-7-induced cell death was more intense in M2 cells than in M1 macrophages under caspase-compromised conditions at all investigated doses (Fig. 2b). Under the same experimental settings, birinapant treatment did not result in higher cell death intensity in M2 than in M1 macrophages at any dose. Birinapant-induced cell death was even slightly, but nonsignificantly, more intense in M1 cells (Fig. 2c). We checked whether the detected cell death in caspase-inhibited conditions is due to necroptosis. For this, we pretreated the polarized macrophages with a specific RIPK1 inhibitor Necrostatin-1 (Nec-1) and RIPK3 inhibitor (GSKʹ872). We observed that Nec-1 and GSKʹ872 blocked birinapant/Z-VAD (BZ)-induced cell death equally in the two cell types. In the case of M2 cells, Nec-1 and GSKʹ872 also inhibited the 5Z-7/Z-VAD-induced cytotoxicity (Fig. 2d). We also analyzed the phosphorylation of MLKL following BZ and 5Z-7/Z-VAD activation as a characteristic marker of necroptosis. We detected phospho-MLKL upon BZ activation in both M1 and M2 cells, but its phosphorylation occurred only in M2 macrophages following 5Z-7/Z-VAD stimulation (Fig. 2e). TNF-R1: Fc fusion protein only partially, but not significantly inhibited 5Z-7/Z-VAD or BZ -induced necroptosis, indicating that other mechanisms than autocrine TNF production may also play a role in 5Z-7/Z-VAD- or BZ-induced necroptosis in human macrophages (data not shown).

Altogether, M2 macrophages are sensitive, but M1 cells are intrinsically resistant to 5Z-7/Z-VAD-induced necroptosis, whereas all other examined stimuli caused the same intense cell death in the two differently polarized macrophage populations.

### Co-culturing M1 and M2 cells does not sensitize M1 cells to TAK1 inhibitor-induced necroptosis

**Fig. 3:**
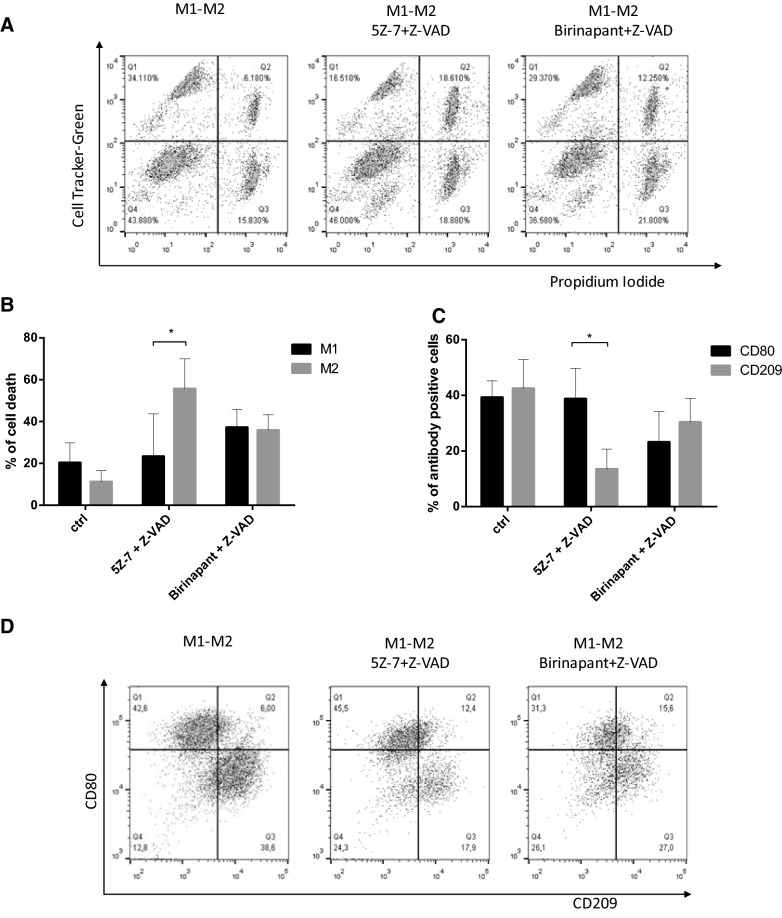
TAK1 inhibitor induces necroptosis on M2, but not on M1 cells in the co-culture of macrophage populations. **a**, **b** M2 cells were loaded with 10 ng/ml CellTracker™ Green CMFDA Dye for 30 min. Green labeled M2 cells and M1 cells were mixed and the cells were treated with 0.5 μM birinapant or 1 μM 5Z-7-oxozeaenol with 50 μM Z-VAD. **a** The degree of total cell death was quantified based on the uptake of PI. **b** A representative image of five independent experiments is documented. Percentage of cell death was calculated in the CellTracker positive and CellTracker negative populations. Figures show the mean plus SD of at least five independent experiments. **c**, **d** M1 and M2 cells were co-cultured and the cells were treated with 0.5 μM birinapant or 1 μM 5Z-7-oxozeaenol in the presence of 50 μM Z-VAD for 24 h. **c** The cell surface expression of CD80 and CD209 were measured by flow cytometry before and after 24 h the indicated treatments. The living cells were gated and the percentage of CD80 positive CD209 negative (CD80) and CD209-positive CD80 negative (CD209) cells were determined. Figure shows the mean plus SD of at least five independent experiments. **d** The cell surface expression of CD80 and CD209 were measured by flow cytometry before or 24 h after the indicated treatments. A representative image of five independent experiments is shown

The two different macrophage phenotypes are present simultaneously at the site of chronic inflammation or in the tumor microenvironment. We tested the ability of the two cell types to regulate each other’s sensitivity to necroptosis by co-culturing M1 and M2 cells. We loaded M2 macrophages with CellTracker™ Green CMFDA Dye and co-cultured cells were treated with BZ or 5Z-7/Z-VAD for 24 h. We determined the intensity of cell death in both CellTracker-positive and -negative populations. Consistent with the results observed with the separately treated cell types, M2 cells were sensitive, but M1 cells remained resistant to 5Z-7/Z-VAD-induced necroptosis. In contrast, BZ treatment effectively killed both macrophage populations in the co-culture (Fig. 3a, b). We confirmed this result by measuring the cell surface markers of the surviving cell populations before and after the induction of cell death. 5Z-7/Z-VAD treatment reduced the amount of CD209 positive cells and consequently pushed the balance of M1/M2 cells toward M1 excess, while BZ treatment had no significant effect on the M1/M2 ratio (Fig. 3c, d). Overall, the treatment of co-cultured M1 and M2 macrophages with TAK1 inhibitor shifted the balance of surviving cells toward M1 dominance. Based on these results, we can conclude that the difference in the sensitivity of the two macrophage subtypes to necroptosis does not depend on M2-derived cytotoxic or M1-derived survival factors, but is regulated by the intrinsic properties of the two cell subtypes.

### The inhibition of the downstream components of TAK1 signaling induces necroptosis in M2 cells

**Fig. 4:**
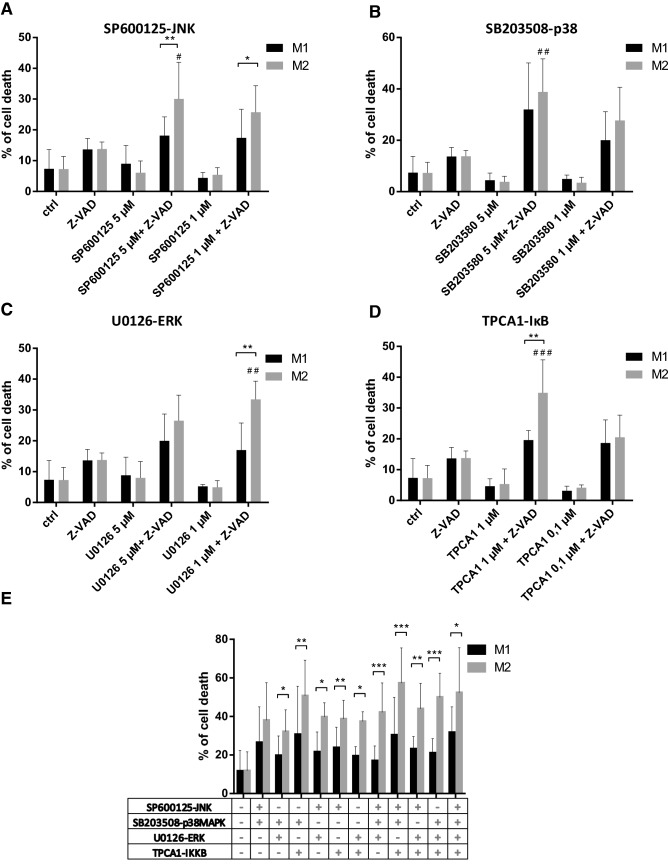
p38MAPK, JNK, ERK and IKK inhibitors induce cell death in M2 macrophages. The in vitro differentiated human M1 and M2 cells were stimulated in the presence or absence of 50 μM Z-VAD for 24 h with the indicated dose of **a** SP600125 (JNK inhibitor), **b** SB203508 (p38MAPK inhibitor), **c** U0126 (ERK inhibitor) and **d** TPCA1 (IKK inhibitor) or **e** with the combinations of these inhibitors. The degree of total cell death was quantified based on the uptake of PI. Figures show the mean plus SD of at least five independent experiments. # represents significant differences to the Z-VAD treated sample

TAK1 regulates the activation of mitogen-activated protein kinases (MAPKs) and nuclear factor kappa B (NFkB) signaling. We attempted to find which downstream component of TAK1 signaling could be responsible for TAK1 inhibitor-induced necroptosis. For this purpose, molecules in TAK1-regulated pathways were inhibited one by SP600125 (JNK inhibitor), SB203508 (p38MAPK inhibitor), U0126 (ERK inhibitor) and TPCA1 (IKK inhibitor). Under caspase-compromised conditions, all these inhibitors induced cell death in M2 macrophages. When we used suboptimal doses of these inhibitors, we detected more intense cell death in M2 than in M1 cells. All observed differences were significant, except for p38 kinase inhibitors (Fig. 4a–d). Caspase activity protected both macrophage subtypes from MAPK or IKK inhibition-induced necroptosis. Combinations of these inhibitors also induced more intense cell death in M2 than in M1 macrophages (Fig. 4e). Phorbol 12-myristate 13-acetate (PMA) treatments resulted in comparable intense phosphorylation of MAPKs and IκB in M1 and M2 cells, indicating that the observed differences in cell death appear to be due to alterations in the necroptotic pathway, and not due to the availability of MAPK signaling (Figure Supplementary S1). According to our observations, we concluded that the absence of any survival signals results in the necroptosis of M2 cells under caspase-compromised conditions, but presumably M1 macrophages utilize additional survival signals to block TAK1 inhibition-mediated necroptosis.

### Inhibitors of Aurora kinase A increased the sensitivity of M1 cells to TAK1 inhibitor-induced necroptosis

**Fig. 5:**
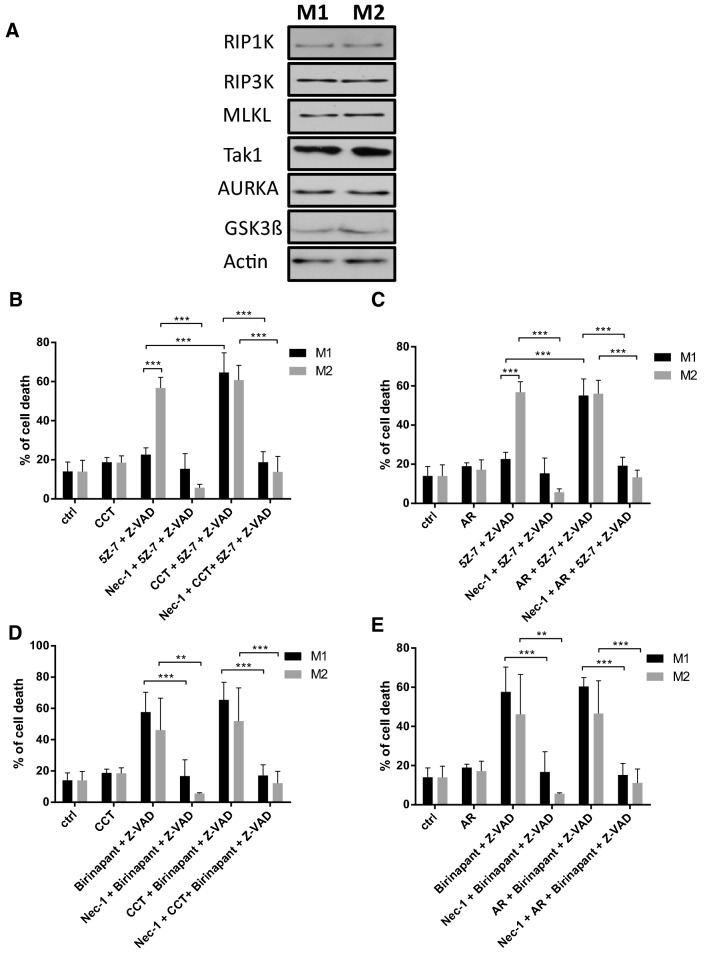
Aurora kinase A inhibitor restores the TAK1 inhibitor-induced cell death in M1 macrophages. **a** The expressions of the indicated molecules were visualized by western blotting of total cell lysates in the in vitro differentiated human M1 and M2 cells. A representative image of three independent experiments is documented. **b**, **c** Macrophages were pre-treated with 1.25 μM CCT137690 (AURKA inhibitor) and 10 μM AR-A014418 (GSK3β) inhibitor, 38.5 μM Necrostatin-1 for 1 h followed by activation with 1 μM 5Z-7-oxozeaenol together with 50 μM Z-VAD. **d**, **e** Macrophages were pre-treated with 1.25 μM CCT137690 (AURKA inhibitor) and 10 μM AR-A014418 (GSK3β) inhibitor, 38.5 μM Necrostatin-1 for 1 h followed by activation with 0.5 μM birinapant together with 50 μM Z-VAD. **b**–**e** After 24 h the extent of cell death was determined by measuring the PI staining. The figure shows the mean plus SD of at least five independent experiment

To explore the molecular background behind the different sensitivities of M1 and M2 cells to necroptosis, we checked the protein expression of necrosome components in M1 and M2 cells. However, we could not detect considerable differences in RIPK1, RIPK3 or MLKL expression (Fig. 5a). Because our results suggested the existence of an extra survival signal in M1 cells, we focused on the inhibitors of necroptosis. AURKA has been recently identified as an inhibitor of necroptosis, which binds to RIPK3 and/or RIPK1 in resting cells and blocks the assembly of the necrosome [30]. GSK3β kinase was identified as the downstream mediator of AURKA in the downregulation of necroptosis. AURKA inhibitor (CCT137690) and GSK3β inhibitor (AR-A014418) have been shown to induce necroptosis in the PDAC cell line [30]. Our results show that treatment with CCT or AR had only minimal effect on macrophage survival under caspase-compromised conditions. Co-administration of BZ with these inhibitors did not significantly change the effect of BZ-induced necroptosis on either M1 or M2 cells (Fig. 5d, e). Importantly, cell death of M1 cells was significantly higher upon 5Z-7/Z-VAD stimulus in the presence of either CCT or AR (Fig. 5b, c). To check the modality of cell death detected in the presence of CCT and AR, we pretreated the polarized macrophages with Nec-1 or GSKʹ872. We observed that Nec-1 and GSKʹ872 completely blocked the BZ-CCT- or BZ-AR-induced cell death (Fig. 5d, e, Figure Supplementary S2) and Nec-1 and GSKʹ872 also completely inhibited the 5Z-7/Z-VAD-CCT- or 5Z-7/Z-VAD-AR-induced cell death in M1 and M2 cells (Fig. 5b, c, Figure Supplementary S2).

Because CCT or AR had no effect on 5Z-7/Z-VAD-induced necroptosis on M2 cells, AURKA or GSK3β inhibitors rendered the two macrophage populations equally sensitive to TAK1 inhibitor-induced necroptosis. We checked the protein expression of AURKA and GSK3β in M1 and M2 cells, but could not detect any differences. These results indicate that the AURKA- or GSK3β-mediated survival pathway, but not the expression level of these kinases, may differ between M1 and M2 cells (Fig. 5a).

### TAM-like macrophages are sensitive to TAK1-inhibitor-induced necroptosis

**Fig. 6:**
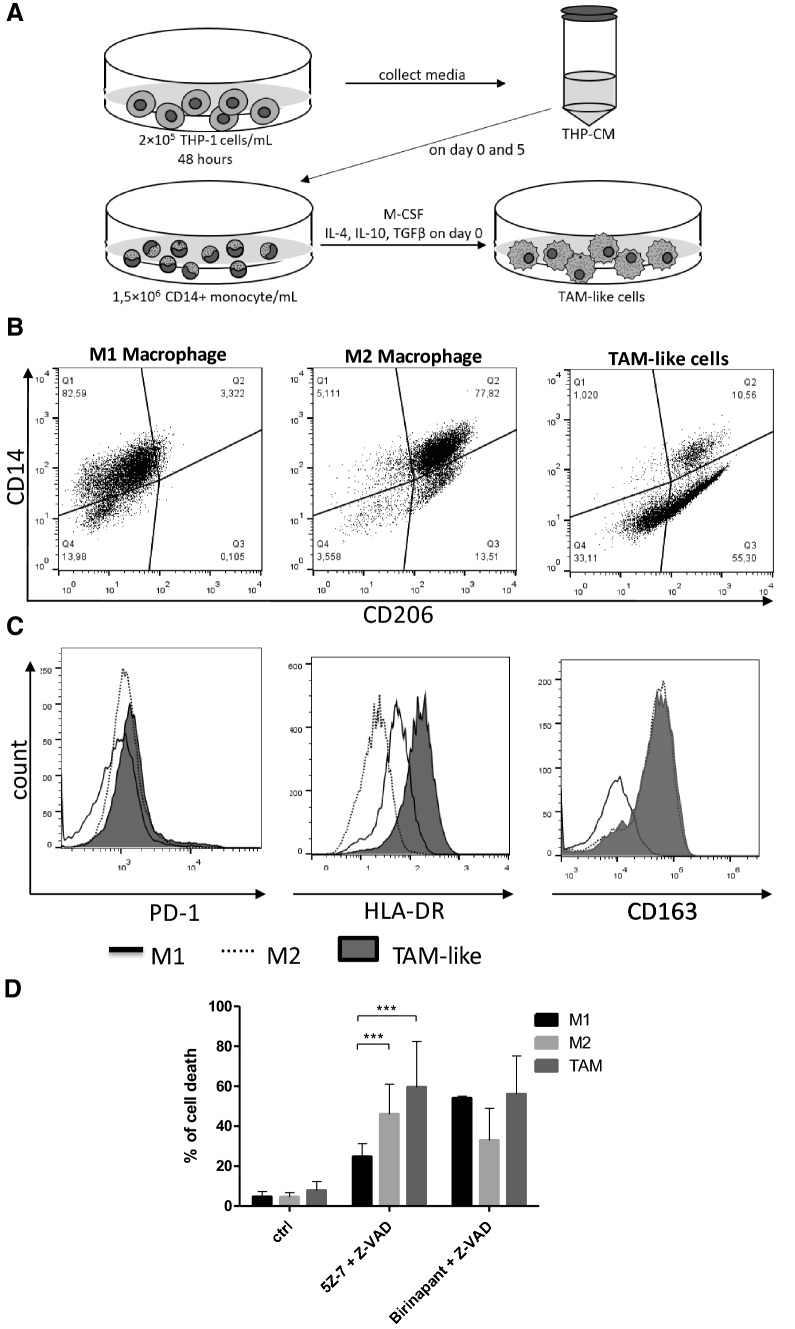
TAK1 inhibitor induces necroptosis on TAM-like macrophages. **a** Schematic of the generation of TAM-like cells. Medium was conditioned on THP-1 cells for 48 h, and was subsequently collected and centrifuged to remove the THP-1 cells (THP-CM). CD14+ monocytes were harvested from healthy donors and plated at 1.5 × 10^6^ cells/ml. During TAM-like cell differentiation process, M-CSF, IL-4, IL-10 and TGFβ cytokines were added to the culture for 5 days in THP-CM. On day 5 the medium was changed to fresh THP-CM. On day 6 the TAM-like cells were harvested. **b**, **c** In vitro generated TAM-like cells were compared to classically and alternatively activated macrophages. Cells were stained and measured on day 6 by flow cytometry to assess surface expression of CD14 and CD206, or CD163, PD-1 and HLA-DR. Representative flow plots and histograms are shown. **d** The in vitro differentiated human M1, M2 and TAM-like cells were treated with 0.5 μM birinapant or 1 μM 5Z-7-oxozeaenol with 50 μM Z-VAD. Cell death was quantified by PI staining in 24 h. Figure shows the mean plus SD of at least five independent experiments

Based on our observations (Fig. 2b), monocyte-derived M2 cells are more prone to TAK1 inhibitor-induced necroptosis than M1 cells. Because the M2-like TAMs show similar functional properties to M2 macrophages, TAMs were in vitro differentiated and the cell death intensities of these TAM-like cells were compared to M1 cells (Fig. 6a–c). Isolated monocytes were plated in medium complemented with M-CSF and IL-10, IL-4 and TGFβ and the supernatant of THP-1 cells. In vitro differentiated TAM-like cells were characterized by flow cytometry. In good accordance with published data [34–36], these cells were CD206 and CD163 positive, but expressed low amounts of CD14 (Fig. 6b, c), while the appearance of MCHII [37] and PD-1 [38] was more intense on the surface of TAM-like cells compared to M1 and M2 macrophages (Fig. 6c). The differentiated TAM-like cells were treated with BZ and 5Z-7/Z-VAD to induce necroptosis. We found that TAM-like cells were as sensitive to BZ-induced necroptosis as M1 cells, but significantly more susceptible to 5Z-7/Z-VAD treatments than M1 macrophages (Fig. 6d). Sensitivity of TAM-like macrophages to TAK1-inhibitor-induced necroptosis promises to be an effective therapeutic strategy to eliminate immunosuppressive macrophages, while preserving the inflammatory M1 cells in the tumor microenvironment.

## Discussion

Due to the heterogenicity of macrophages, dramatic functional differences have been observed depending on their polarization. The two extremes in the spectrum are M1 macrophages, which are responsible for the initiation of inflammation, and alternatively activated M2 macrophages with anti-inflammatory properties. Targeted modulation of M1/M2 transition may offer promising strategies to cure various life-threatening diseases. Accordingly, ongoing clinical trials aim to modulate M1/M2 balance in cancer [5, 6], atherosclerosis [2], sclerosis multiplex [39] or endometriosis [40]. Currently, the therapeutic approaches to alter the ratio of macrophage subsets are bipartite, on the one hand directing the differentiation of these cells, and on the other hand, altering the function of the differentiated cells [6]. In addition, targeted depletion of each macrophage type also offers a therapeutic option to regulate the M1/M2 ratio. This strategy can be relevant, especially in TME, where cell death induction is certainly the main therapeutic intervention [6]. Necroptotic stimuli, among them SMs [41] and TAK1 inhibitors [42], have been tested in various ongoing clinical trials to eliminate apoptosis-resistant tumor cells. However, necroptosis induction in the TME is a double-edged sword, because necroptosis in the TME can also result in immunosuppression, and necroptotic cell death of endothelial cells promotes tumor cell extravasation and metastasis [18]. Consequently, the effect of any necroptosis inducers should be checked also on the TME in addition to its tumor-killing capacity. The effect of SM and TAK1 inhibitors on the cellular components of tumor-associated stroma is not clearly investigated.

Macrophages are relatively resistant to apoptosis, but are highly sensitive to necroptosis [43]. SMAC mimetics have been reported to trigger cell death in macrophages, especially under caspase-compromised conditions [31], and TAK1 inhibitors are also well known to induce necroptosis in macrophages [14].

We attempted to compare the sensitivity of M1 and M2 macrophages to various cell death stimuli. The two macrophage populations were equally sensitive to most of the investigated apoptotic or necroptotic inductions, but we observed that monocyte-derived M2 cells are more prone to TAK1 inhibitor-induced necroptosis than M1 cells. Because the M2-like TAMs generally show similar functional properties as M2 macrophages, the question arose as to whether these two anti-inflammatory populations use similar cell death pathways. We generated TAM-like cells in vitro by using the traditional M2 differentiation protocol and THP-1 conditioned media. TAM-like macrophages, as well as M2 cells, were sensitive to TAK1 inhibitor-induced necroptotic stimuli. The difference in the sensitivity of M1 and M2 macrophages to TAK1 inhibitor-induced necroptosis was still observed when the two cell populations were co-cultured, which results in exclusion the possibility that the effect is due to an autocrine cytotoxic factor exclusively produced by M2 cells. Our findings suggest that TAK1 inhibitors are more promising candidates for tumor therapy than SM due to the intense killing of anti-inflammatory macrophages. However, further investigations are needed to check the effect of the two necroptotic treatments on other suppressive cell types in the TME, and in vivo studies should also be performed to investigate the recruitment of various cells following treatments with both TAK1 inhibitor and SM. The use of TAK1 inhibitor instead of SM is rationalized not only by the susceptibility of M2- and TAM-like macrophages to TAK1 inhibitor-induced necroptosis but also by the fact that TAK1-mediated events also have been demonstrated in all growth factor signaling, Treg cell development, epithelial–mesenchymal transition, angiogenesis and in resistance to conventional chemotherapy [42].

Investigation of the intrinsic signaling pathways of the two cell types indicates that any perturbation in the survival signals resulted in necroptosis in M2 cells, but M1 cells were relatively unaffected. The higher degree of resistance indicates the existence of a special survival signal in M1 cells. AURKA acts as a local inhibitor against spontaneous necroptosis, by associating with RIPK3 and RIPK1 in resting cells [30]. In the presence of AURKA inhibitors, M1 cells became as sensitive to TAK1 inhibitor-induced cell death as the M2 cells were. GSK3β was identified as the downstream target of AURKA in the regulation of necroptosis [30], and consequently, GSK3β inhibitors also restored the sensitivity of M1 cells to TAK1 inhibitor-induced cell death. AURKA inhibitors are also intensively studied in tumor therapy [44], but their effect on TME is less intensively investigated. Hereby, we highlight that AURKA may have an effect on the immunosuppressive microenvironment, keeping M1 cells alive, which can be considered in therapeutic approaches using AURKA inhibitors either alone or in co-therapy.

More than 20 drugs, approved mostly in cancer therapy, but also in autoimmune or neurodegenerative disorders, have the potential to regulate necroptosis [18]. How these drugs modify the life span of M1/M2 cells still have not been investigated, but this effect may have influence on the current applications of necroptosis regulators.

Two different types of necroptosis have been detected by TNF induction in 661 W mouse photoreceptor cell lines. Activation of TNF/5Z-7/Z-VAD resulted in different necroptosis than TNF/CHX/Z-VAD concerning the molecular composition and also the signal transduction of necroptotic pathway [45].

Our results indicate heterogeneous functionality of different necroptotic stimuli, highlighting the importance of specifying and differentiating necroptotic pathways. Overall, our findings provide new approaches to regulate the balance of M1/M2 cells in the treatment for various diseases such as cancer or chronic infections.

## Electronic supplementary material

Below is the link to the electronic supplementary material.

Supplementary material 1 (PDF 280 kb)

Supplementary material 2 (PDF 441 kb)
