# Supplementary material for: Differences in the sensitivity of classically and alternatively activated macrophages to TAK1 inhibitor-induced necroptosis
Source: Cancer Immunol Immunother. 2020 May 29;69(11):2193–207. doi: 10.1007/s00262-020-02623-7 (PMC7568718; doi:10.1007/s00262-020-02623-7)
Supplement: Supplementary file 1 — Supplementary material 1 (PDF 280 kb) [file 262_2020_2623_MOESM1_ESM.pdf]

Supplementary figure legends

Supplementary Figure1: Phorbol 12-myristate 13-acetate treatments induces phosphorylation of MAPKs and IκB in M1 and M2 cells.

In vitro differentiated human M1 and M2 cells were activated by 100 nM Phorbol 12-myristate13-acetate (PMA) for the indicated times. Phosphorylation of p38 MAPK, JNK, ERK and IKK were visualized by western blotting of total cell lysates using phospo-specific antibodies. A representative image of three independent experiments is shown.

Supplementary Figure 2: Aurora Kinase A inhibitor restores the TAK1 inhibitor-induced cell death in M1 macrophages.

(A-B) Macrophages were pre-treated with 1.25 µM CCT137690 (AURKA inhibitor), 10 µM AR-A014418 (GSK3β) inhibitor, 7,5 µM GSK′872 RIPK3 inhibitor and for 1 hour followed by activation with 1 µM 5Z-7-oxozeaenol together with 50 µM Z-VAD). (C-D) Macrophages were pre-treated with 1.25 µM CCT137690 (AURKA inhibitor), 10 µM AR-A014418 (GSK3β) inhibitor, 7,5 µM GSK′872 RIPK3 inhibitor and for 1 hour followed by activation with 0.5 µM birinapant together with 50 µM Z-VAD. (A-D) After 24 hours the extent of cell death was determined by measuring the PI staining. The figures show the mean plus SD of at least five independent experiment.
