# Supplementary figures and images for: Differences in the sensitivity of classically and alternatively activated macrophages to TAK1 inhibitor-induced necroptosis
Source: Cancer Immunol Immunother. 2020 May 29;69(11):2193–207. doi: 10.1007/s00262-020-02623-7 (PMC7568718; doi:10.1007/s00262-020-02623-7)

# Supplementary 1

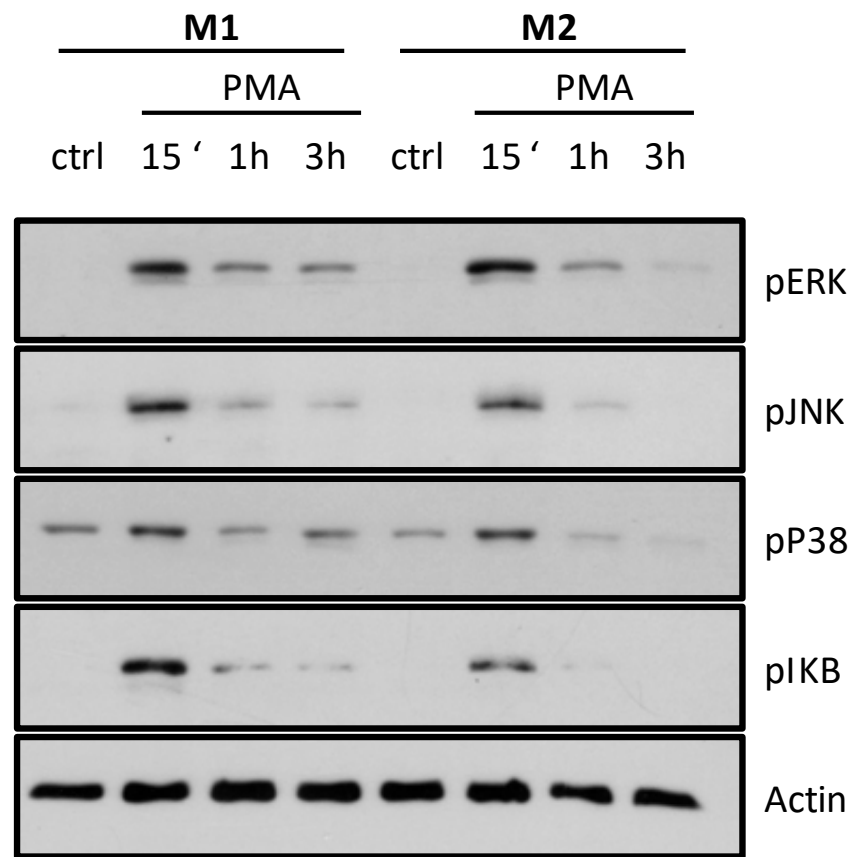

**A**

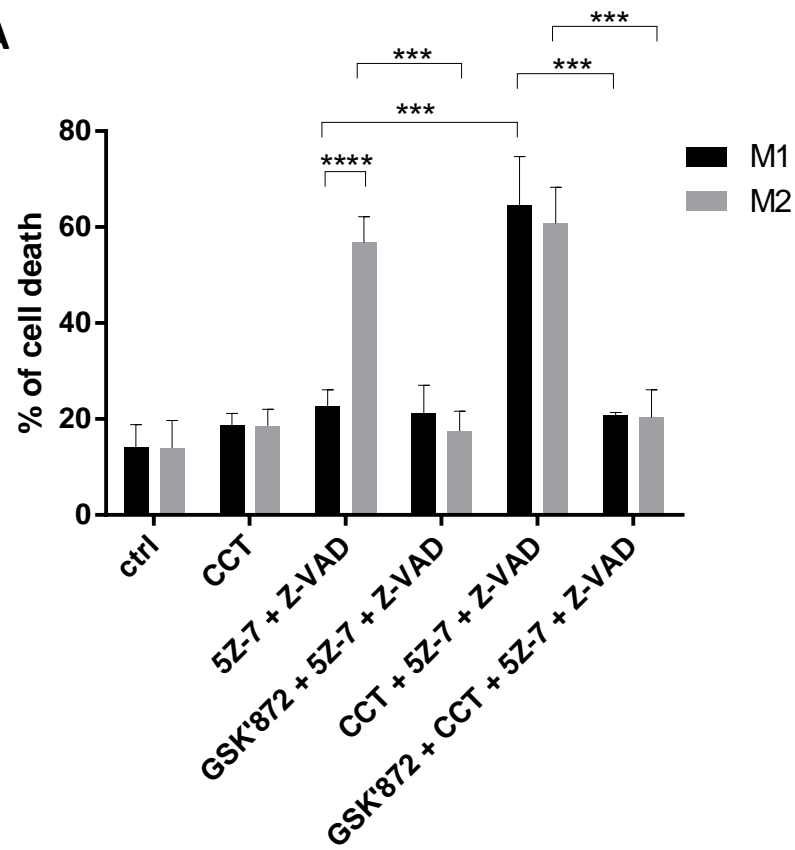

**B**

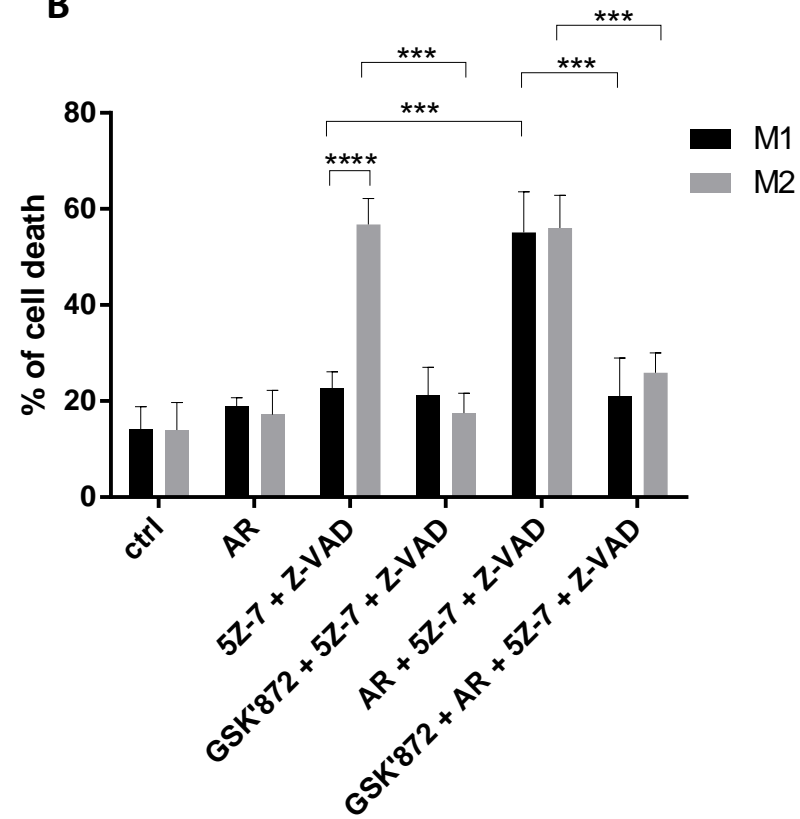

**C**

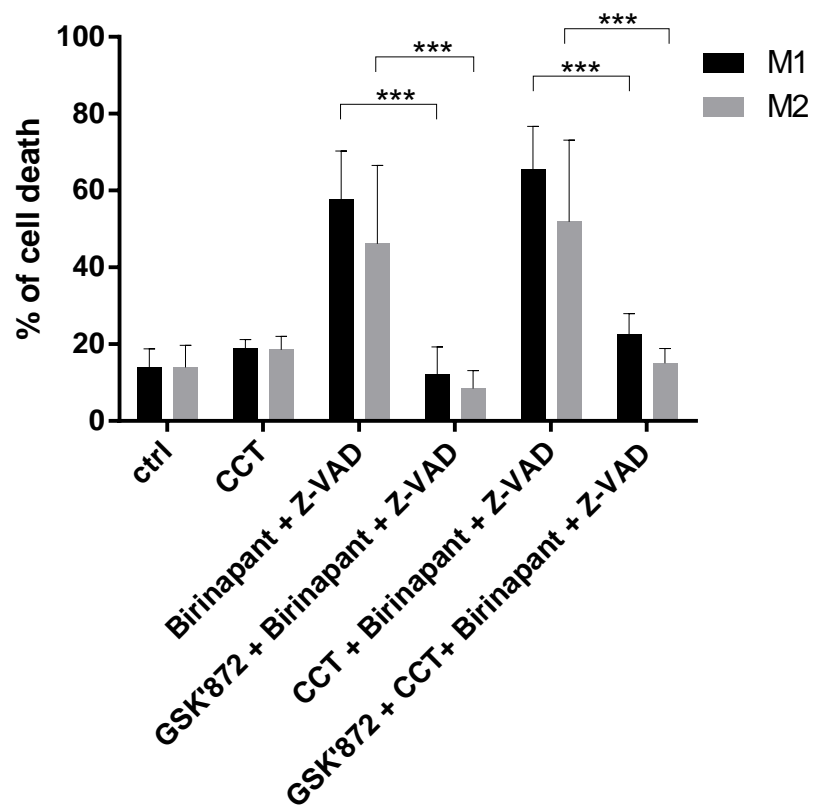

**D**

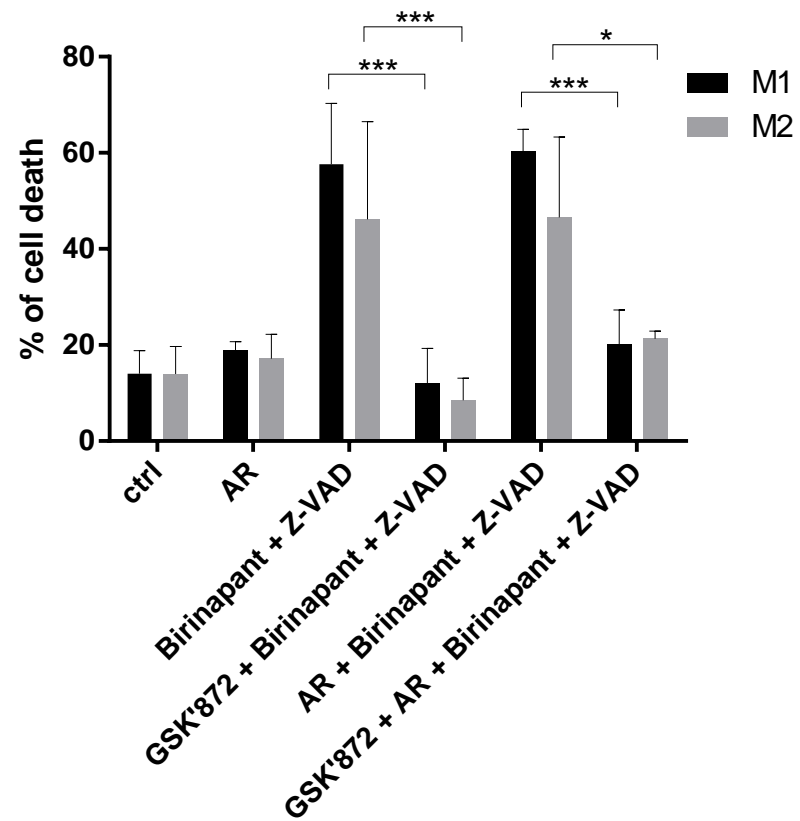

Supplement: Supplementary file 2 — Supplementary material 2 (PDF 441 kb) [file 262_2020_2623_MOESM2_ESM.pdf]
